# Expression of a Crry/p65 is reduced in acute lung injury induced by extracellular histones

**DOI:** 10.1002/2211-5463.13322

**Published:** 2021-11-08

**Authors:** Fumihiko Nagano, Tomohiro Mizuno, Masaki Imai, Kazuo Takahashi, Naotake Tsuboi, Shoichi Maruyama, Masashi Mizuno

**Affiliations:** ^1^ Department of Nephrology Nagoya University Nagoya Japan; ^2^ Department of Analytical Pharmacology Meijo University Nagoya Japan; ^3^ Department of Clinical Pharmacy Fujita Health University Toyoake Japan; ^4^ Department of Immunology Nagoya City University Nagoya Japan; ^5^ Department of Cell Biology and Anatomy Fujita Health University Toyoake Japan; ^6^ Department of Nephrology Fujita Health University Toyoake Japan; ^7^ Department of Renal Replacement Therapy Nagoya University Nagoya Japan

**Keywords:** acute lung injury, C3a, C3a receptor antagonist, complement receptor type 1‐related gene Y, endothelial cell, extracellular histone

## Abstract

Acute lung injury (ALI) occurs in patients with severe sepsis and has a mortality rate of 40%–60%. Severe sepsis promotes the release of histones from dying cells, which can induce platelet aggregation, activate coagulation and cause endothelial cell (EC) death. We previously reported that the expression of membrane complement receptor type 1‐related gene Y (Crry)/p65, which plays a principal role in defence against abnormal activation of complement in the blood, is reduced in response to peritoneal mesothelial cell injury, and we hence hypothesized that a similar mechanism occurs in pulmonary ECs. In this study, we examined the role of Crry/p65 in histone‐mediated ALI using an experimental animal model. In ALI model mice, exposure to extracellular histones induces lung injury and results in a decrease in Crry/p65 expression. The levels of lactic acid dehydrogenase (LDH), a marker of cell damage, were significantly increased in the serum of ALI model compared with vehicle mice. The significant inverse correlation between the expression of Crry/p65 and LDH levels in plasma revealed an association between Crry/p65 expression and cell damage. The levels of complement component 3a (C3a) were also significantly increased in the serum of the ALI model compared with vehicle mice. Notably, a C3a receptor antagonist ameliorated lung injury induced by histones. We hypothesize that extracellular histones induce complement activation via down‐regulation of Crry/p65 and that C3a might serve as a therapeutic target for the treatment of ALI.

## Graphical abstract

Extracellular histones decrease the expression of complement receptor type 1‐related gene Y (Crry)/p65 in an experimental model of acute lung injury (ALI). Dysfunction of Crry/p65 is associated with endothelial damage and promotes C3a production resulting from complement activity. C3a is a potential therapeutic target for ALI.

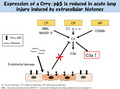

**Abbreviations**
ALI: acute lung injury
APC: allophycocyanin
APTT: activated partial thromboplastin time
BV: Brilliant violet
C: complement
C3a: complement component 3a
Crry: complement receptor type 1‐related gene Y
DMSO: dimethyl sulfoxide
EC: endothelial cell
EDTA: ethylenediaminetetraacetic acid dipotassium salt
ELISA: enzyme‐linked immunosorbent assay
FBS: fetal bovine serum
FITC: fluorescein isothiocyanate
LDH: lactic acid dehydrogenase
MS: mouse serum
PE: phycoerythrin
PT: prothrombin time

Acute lung injury (ALI) occurs in patients with severe sepsis and has a mortality rate of 40%–60% [1, 2, 3, 4]. Severe sepsis promotes the release of histones from dying cells, and these basic proteins can form reversible complexes with DNA [5, 6]. Histones derived from dying cells also induce platelet aggregation, activate coagulation [7] and cause endothelial cell (EC) death [8]. In particular, impairment of the alveolar–capillary barrier function, resulting from endothelial damage, induces complex responses in the lungs [6]. These complex responses include the promotion of the tendency for alveolar flooding, subsequently inducing pulmonary oedema by impairing fluid clearance [9, 10]. In experimental animal models, extracellular histones induce ALI characterized not only by pulmonary oedema but also by bleeding. Although pulmonary oedema and bleeding reflect clinical symptoms, the detailed mechanisms underlying these responses are currently unclear.

Vascular ECs are consistently exposed to the flow of blood, and to protect the vasculature against abnormal complement (C) activity, they function as C regulators [11, 12, 13, 14, 15]. Their barrier function is based on the activity of membrane C regulators, namely CD46, CD55 and CD59. In particular, CD46 regulates C activation at the C3 level on the surface of ECs. In murine species, complement receptor type 1‐related gene Y (Crry)/p65 binds to C3b and C4b, and shows the same inhibitory activity as human C receptor 1 [16] and factor I cofactor. Because Crry/p65 possesses functions of both CD46 and CD55, it provides a more potent inhibition of classical and alternative pathways in rodents [16, 17, 18].

The findings of previous studies have indicated that anti‐C therapy can ameliorate ALI in experimental models [19, 20, 21]; however, there is currently limited information regarding C regulators in ALI induced by extracellular histones. Extracellular histones can induce EC damage and apoptosis [8, 22, 23], leading to changes in membrane conformation. We previously reported that the expression of membrane Crry/p65 was reduced in response to peritoneal mesothelial cell injury [24], and we hence assumed that a similar mechanism occurs in pulmonary ECs. In the present study, we sought to elucidate the roles of Crry/p65 in ALI by investigating whether extracellular histones decrease the expression of Crry/p65 in an experimental animal model.

## Materials and methods

### Reagents and antibodies

We used the following reagents: RPMI medium (Sigma‐Aldrich, St Louis, MO, USA), fetal bovine serum (FBS; Thermo Fisher Scientific, Waltham, MA, USA), unfractionated histones from calf thymus (Sigma‐Aldrich), ethylenediaminetetraacetic acid dipotassium salt (EDTA‐2K; Dojindo Laboratories, Kumamoto, Japan), saline (Otsuka Pharmaceutical, Tokyo, Japan), sodium citrate (Sigma‐Aldrich), 0.4% trypan blue and trypsin/EDTA solutions (Thermo Fisher Scientific), paraformaldehyde phosphate buffer solution (Wako, Osaka, Japan), Hoechst 33342 solution (Dojindo Laboratories), 0.5% eosin Y ethanol solution (Wako), Mayer's haematoxylin solution (Wako), OCT compound (Sakura Fine Technical, Tokyo, Japan), Thrombocheck PT and Thrombocheck APTT (Sysmex, Kobe, Japan), AutoMACS Running Buffer and Multi‐Tissue Dissociation Kit 1 (Miltenyi Biotec, Bergisch Gladbach, Germany), a mouse complement component 3a (C3a) enzyme‐linked immunosorbent assay (ELISA) kit (Novus Biologicals, Englewood, CO, USA) and SB 290157 (a C3a receptor antagonist; EMD Millipore, Burlington, VT, USA).

Antibody sources were as follows: Brilliant violet (BV)421‐conjugated rat IgG2a anti‐mouse Crry/p65 (clone 1F2; BD Biosciences, San Jose, CA, USA), BV421‐conjugated rat IgG2a isotype control (clone RTK2758; BioLegend, San Diego, CA, USA), allophycocyanin (APC)‐conjugated rat IgG2a anti‐mouse CD31 (clone 390; BioLegend), APC‐conjugated rat IgG2a isotype control (clone RTK2758; BioLegend), phycoerythrin (PE)‐conjugated rat IgG2b anti‐mouse CD45 (clone 30‐F11; BioLegend), PE‐conjugated rat IgG2b isotype control (clone RTK4530; BioLegend) and fluorescein isothiocyanate (FITC)‐conjugated rabbit polyclonal IgG anti‐C3c (Abcam, Cambridge, UK).

### Animal model of ALI

All animal experiments in the present study were approved by the Experimental Animal Board of Meijo University (approval number 2018‐PE‐31) and Nagoya University (approval number 20353). Male C57BL/6J mice (Japan SLC, Shizuoka, Japan; age 9–12 weeks) were used for the ALI animal model. The mice were maintained under conventional laboratory conditions and were provided free access to food and water. The ALI animal model was prepared according to the procedure described in previous studies [25, 26, 27]. The mice received a single tail vein injection of calf thymus histones (30–60 μg·g^−1^ body weight) or saline, and lung samples were collected from anaesthetized mice 10 or 30 min later. After weighing, the lung samples were snap‐frozen in OCT compound. To prevent blood coagulation, the samples were mixed with EDTA‐2K or 3.13% (w/v) sodium citrate. To assess the cell damage in the ALI animal model, lactic acid dehydrogenase (LDH) levels in plasma were also measured by Oriental Yeast.

### Histological analysis

Lung tissue sections (10 μm thick) were prepared using a cryostat and stained with haematoxylin and eosin for histological analysis.

To measure the proportion of bleeding area in a tissue section, whole‐section images were captured at 40 × magnification under a BZ‐X700 Fluorescence Microscope (Keyence, Osaka, Japan). The extent of pulmonary haemorrhage, defined as the lung injury area, was measured using bz‐x analysis software (Keyence).

### Immunohistochemistry

Sections of lung tissues were prepared using a cryostat and treated with 10% goat serum for 20 min at room temperature. After washing three times with PBS, the tissues were incubated with rat monoclonal IgG anti‐Crry/p65 (clone 1AF; Becton Dickinson) on ice for 30 min. The tissues were washed three times with PBS, and then incubated with Alexa Fluor 488‐conjugated goat IgG anti‐rat IgG (clone poly4054; Bio Legend) in the dark on ice for 1 h. After washing three times with PBS, the tissues were incubated with Alexa Fluor 594‐conjugated rat monoclonal IgG anti‐mouse CD31 containing Hoechst 33342 solution in the dark on ice for 1 h. The images were captured at 200 × magnification under a BZ‐X700 Fluorescence Microscope (Keyence). To quantify Crry/p65 expression, we measured the brightness of the CD31‐ and Crry/p65‐positive area in the whole section using BZ‐X Analysis software and compared the brightness between vehicle and histone groups.

### Platelet counts and coagulation test

Platelet counts were performed using blood mixed with EDTA‐2K. Custom measurements for platelet counts were performed by Oriental Yeast. Blood mixed with sodium citrate was used for coagulation tests. Plasma was prepared by centrifuging the blood at 1500 ***g*** for 10 min at room temperature. The prothrombin time (PT) and activated partial thromboplastin time (APTT) were measured using standard methods as described previously [26, 27] using a KC1 Delta automatic coagulation analyser in conjunction with an electromechanical clot detection instrument (Trinity Biotech, Bray, Ireland).

### Preparation of single‐cell suspensions from lung tissues

Lung tissues were weighed, then homogenized using Multi‐Tissue Dissociation Kit 1. Single‐cell suspensions were prepared according to the manufacturer’s instructions and a previous report [28]. Briefly, AutoMACS Running Buffer and RPMI medium containing enzymes of the Multi‐Tissue Dissociation Kit were added to a Gentle MACS C tube (Miltenyi Biotec). After cutting lung tissues in the medium, they were incubated for 30 min at 37 °C. The tissues were then homogenized in the Gentle MACS using program B.01. After homogenization, the samples were filtered through 70‐μm strainers, washed with AutoMACS Running Buffer and subsequently centrifuged at 300 ***g*** for 10 min at room temperature. The single‐cell suspension and tissue supernatant were collected after washing and centrifugation.

To evaluate the correlation between the concentration of C3a in plasma and the expression of Crry/p65, plasma samples were prepared by centrifuging the blood at 1500 ***g*** for 10 min at 4 °C. The concentrations of C3a were measured using a mouse C3a ELISA kit according to the manufacturer's instructions.

### Flow cytometry

We measured the expression of Crry/p65 in vascular ECs isolated from histone‐treated mouse lung tissues. The isolation of ECs was conducted according to manufacturer’s instructions and the previous report [28] in Section 2.5. The cell suspension prepared from lung tissue was washed twice with MACS Running Buffer and then incubated in the dark with BV421‐conjugated rat IgG2a anti‐mouse Crry/p65 or isotype control, APC‐conjugated rat IgG2a anti‐mouse CD31 or isotype control, and PE‐conjugated rat IgG2b anti‐mouse CD45 or isotype control for 10 min at 4 °C. The cell suspensions were then washed twice with phosphate‐buffered saline (PBS) containing 0.1% FBS. After washing, the expression of Crry/p65 in CD31^+^CD45^‐^ vascular ECs was analysed using an LSRFortessa™ X‐20 System (BD Biosciences). To elucidate the association between lung oedema and the reduced expression of Crry/p65, we conducted a correlation analysis between lung weight and Crry/p65 expression.

### Endothelial cell permeability assay

To evaluate vascular endothelial permeability after induction by histone, we performed a cell permeability assay using an In Vitro Vascular Permeability Assay Kit (Sigma‐Aldrich) according to the manufacturer’s instructions. In brief, RCB1994 cells were seeded 3.0 × 10^4^ per insert well and incubated until the formation of a cell monolayer. The cells were incubated with RPMI medium (control), calf thymus histone (100 or 200 μg·mL^−1^) for 30 min or mouse TNF‐α (0.1 μg·mL^−1^) for 19 h as a positive control at 37 °C. After removing the cell medium, the cells were incubated with FITC‐dextran in the dark for 20 min at room temperature. Fluorescence was measured using the EnSpire multimode plate reader (PerkinElmer, Waltham, MA, USA; excitation 485 nm, emission 535 nm).

### Trypan blue staining

Mouse ECs (RCB1994) were purchased from the RIKEN BioResource Center (Ibaraki, Japan). The cells were cultured in RPMI medium supplemented with 10% FBS in humidified air containing 5% CO_2_ at 37 °C. Incubation of RCB1994 cells with extracellular histones was conducted as described previously [27]. Under subconfluent conditions, the culture medium was replaced with serum‐free medium containing calf thymus histones (0–200 μg·mL^−1^), and the cells were incubated for 30 min. After incubation in the presence of the histones, cell suspensions were prepared by incubating with a trypsin/EDTA solution. To measure the percentages of cells that survived, trypan blue solution was added to an equal volume of the cell suspension, and the numbers of dead cells were counted using a TC‐20 automatic cell counter (Bio‐Rad, Hercules, CA, USA). The percentages of surviving cells were calculated as the number of trypan blue negative cells among the total number of cells × 100. After counting, we determined the correlation between cell survival and the expression of Crry/p65 based on flow cytometry measurements.

### Functional assay of C activation

To evaluate the functional decrease in Crry/p65 in RCB1994 cells, we measured the deposition area of C3c on these cells induced by normal mouse serum (MS). RCB1994 cells were seeded at 5.0 × 10^4^ cells·well^−1^ and incubated with RPMI medium (control) or calf thymus histones (200 μg·mL^−1^) for 30 min at 37 °C. After incubation, the cells were washed twice with PBS, then incubated with 5% normal MS or heat‐inactivated MS for 1 h at 37 °C. Then, the cells were fixed with 4% paraformaldehyde phosphate buffer solution for 10 min at room temperature. The cells were washed twice with PBS, and then, the cells were incubated in the dark with FITC‐conjugated rabbit polyclonal IgG anti‐C3c containing Hoechst 33342 solution for 30 min at room temperature. Heat‐inactivated MS was prepared by incubation for 30 min at 56 °C. To measure the C3c‐positive area per cell, we counted cells and measured the positive area in five randomly selected fields, with images being captured at 200× magnification under a BZ‐X700 Fluorescence Microscope (Keyence). Measurements of the C3c‐positive area in images were performed using ImageXpress MICRO (Molecular Devices, San Jose, CA, USA).

### Treatment with a C3a receptor antagonist

To clarify whether a C3a receptor antagonist could ameliorate pulmonary haemorrhage and oedema, we pretreated histone‐injected mice with the C3a receptor antagonist, SB290157, as described previously [29]. Briefly, mice were randomized prior to receiving an intraperitoneal injection of either C3a receptor antagonist (1 mg·kg^−1^ SB290157; Calbiochem, Darmstadt, Germany) diluted in PBS and dimethyl sulfoxide (DMSO; 1.16%, v/v), or an equal volume of this vehicle 45 min before being administered a single tail vein injection of calf thymus histones (45 μg·g^−1^ body weight). Lung samples, collected from anaesthetized mice 30 min after the histone injections, were snap‐frozen in OCT compound after weighing the tissue.

### Statistical analyses

Normality data are displayed as means ± standard deviation. Non‐normality data are displayed by medians and ranges. To assess the homoscedasticity of the data, we conducted Levene's test between two groups. Then, Student’s *t*‐test or the Welch *t*‐test was used for comparisons of homoscedasticity or nonhomoscedasticity data. The Mann–Whitney *U*‐test was used for comparisons of non‐normality data. Comparisons among multiple groups were performed using an analysis of variance followed by Tukey’s test. Pearson’s correlation analysis was performed to determine correlation coefficients. In all statistical analyses, a two‐tailed *P*‐value of ˂ 0.05 was considered significant. spss v25.0 software (IBM, Chicago, IL, USA) was used for statistical analyses.

## Results

### Extracellular histones induce ALI and perturb coagulation

**Fig. 1:**
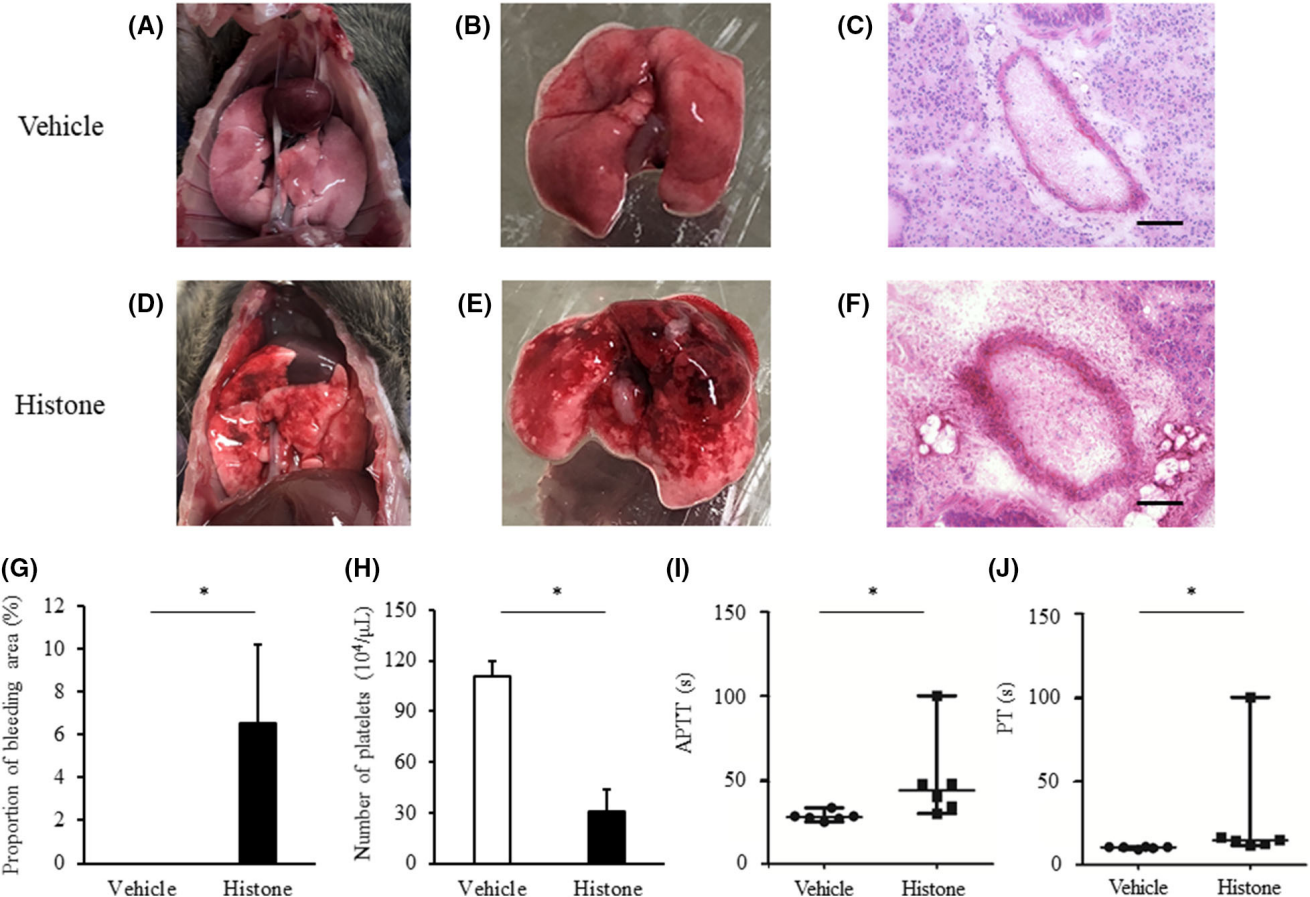
Lung injury, thrombocytopenia, and perturbation of coagulation induced by extracellular histones. C57BL/6J mice received a single tail vein injection of calf thymus histones (45 μg·g^−1^ body weight) or saline (*n* = 6 per group). Panels A to F show representative images of macroscopic (A, B, D, E) and microscopic (C and F) findings. Black bar shows 100 μm. The proportion of bleeding area in a tissue section is shown in panel G. The number of platelets, APTT and PT are shown in panels H–J, respectively. Values are shown as experimental means ± SD (panels G and H) or medians and ranges (panels I and J). *P < 0.01 vs. vehicle (Student’s *t*‐test, panels G and H; or Mann–Whitney U‐test, panels I and J).

To optimize our experiments, we assessed the degree of lung injury at 10, 30 and 60 min after histone injection. Because peak lung injury occurred at 30 min, we used this time for collecting subsequent samples (Fig. S1). To evaluate the severity of lung injury in this experimental model, we performed histological analyses of lung tissues, as well as platelet counts and coagulation tests. After histone injection, severe pulmonary haemorrhage was observed both macroscopically (Fig. 1A,B,D,E) and microscopically (Fig. 1C,F). The area of pulmonary haemorrhage was significantly increased in the histone‐treated group compared with that in the vehicle group (Fig. 1G). Moreover, compared with the vehicle group, there was a reduction in the number of platelets (Fig. 1H) and prolongation of both the APTT (Fig. 1I) and PT (Fig. 1J) in the histone group. Accordingly, these results indicate that histones can induce severe lung injury and coagulation disorder.

### Reduced expression of Crry/p65 is associated with C activation and lung oedema

**Fig. 2:**
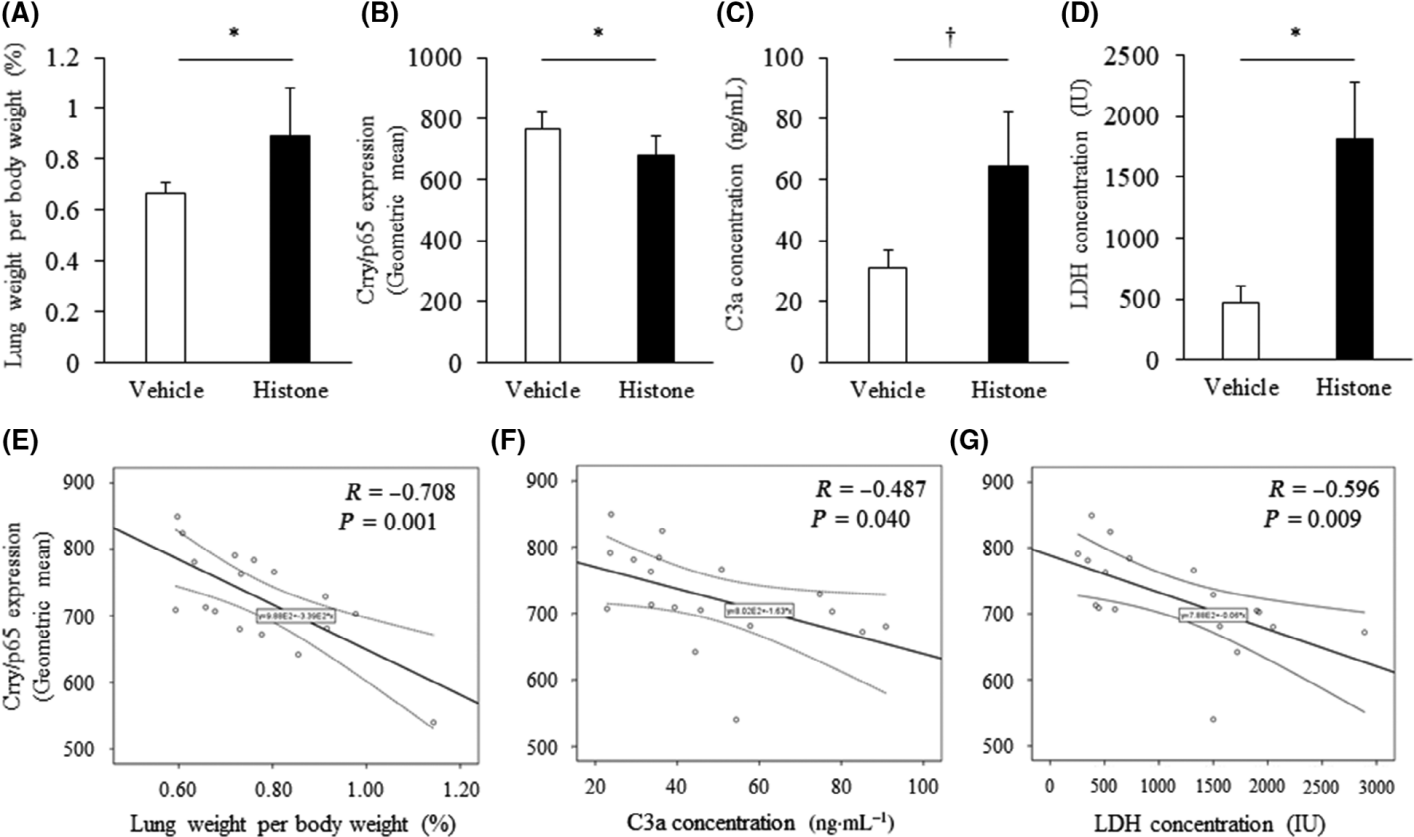
Reduced expression of Crry/p65 associated with lung oedema. C57BL/6J mice received a single tail vein injection of calf thymus histones (45 μg·g^−1^ body weight) or saline (*n* = 9 per group). Lung weight per body weight (%) and Crry/p65 expression are shown in panels A and B, respectively. The correlation between Crry/p65 expression and lung weight per body weight is shown in panel E. The concentrations of C3a in plasma are shown in panel C. The correlation between Crry/p65 expression and C3a in plasma is shown in panel F. The concentrations of LDH in plasma are shown in panel D. The correlation between Crry/p65 expression and LDH in plasma is shown in panel G. Values are shown as experimental means ± SD. *P < 0.01 vs. vehicle (Student's *t*‐test) ^†^ *P* < 0.01 vs. vehicle (Welch’s *t*‐test). Correlation analysis was conducted by Pearson's correlation analysis.

**Fig. 3:**
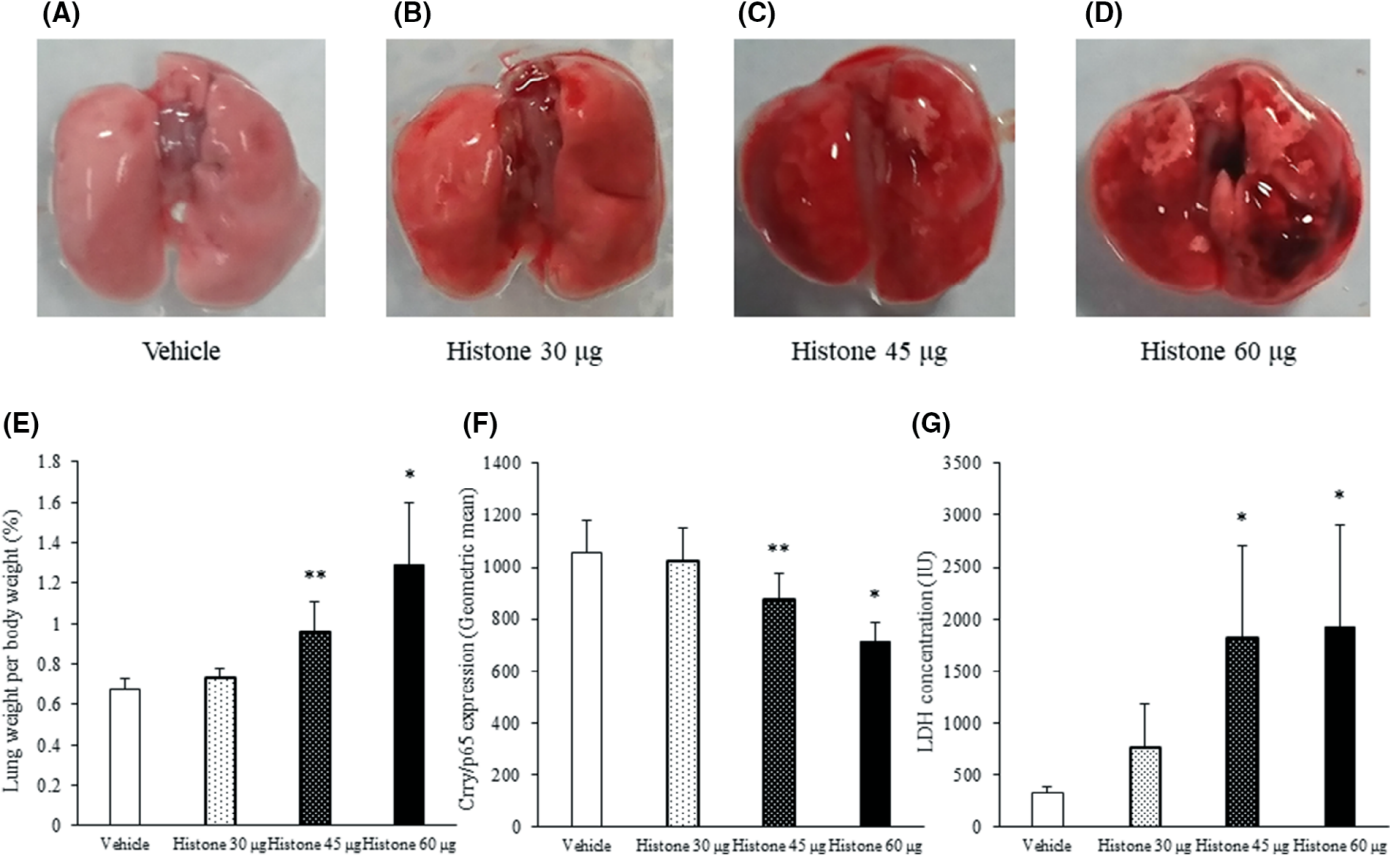
Expression of Crry/p65 was dose‐dependently reduced by extracellular histones. C57BL/6J mice received a single tail vein injection of calf thymus histones (30–60 μg·g^−1^ body weight) or saline (*n* = 8 per group). Panels A–D show the macroscopic findings. Panel E shows the lung weight per body weight (%). Crry/p65 expression and LDH concentration are shown in panels F and G, respectively. Values are shown as experimental means ± SD. **P < 0.05, *P < 0.01 vs. histone (Tukey's test).

**Fig. 4:**
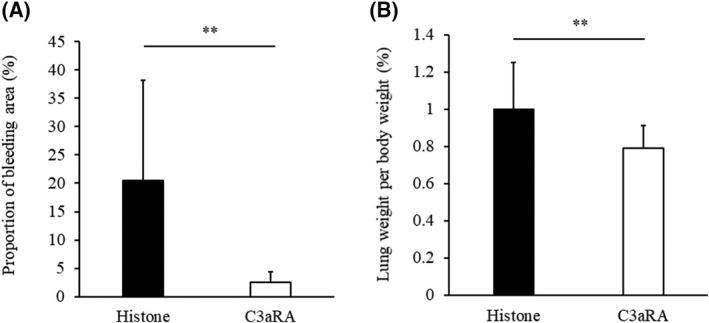
Treatment with C3a receptor antagonist ameliorates lung injury induced by extracellular histones. C57BL/6J mice received a single intraperitoneal injection of C3a receptor antagonist (C3aRA) 45 min prior to histone injection (histone group; *n* = 9, C3aRA group; *n* = 8). Panel A shows the proportion of bleeding area in a tissue section. Lung weight per body weight (%) after histone injection is shown in panel B. Values are shown as experimental means ± SD. **P < 0.05 vs. histone (Student’s *t*‐test).

EC damage initiates pulmonary oedema with impaired fluid clearance [9, 10]. In addition, C activation is known to promote oedema via the production of anaphylatoxins. Thus, we assumed that the promotion of oedema via C activation was associated with the induction of C activation related to Crry/p65 dysfunction. In the present study, Crry/p65 was expressed in lung ECs (Fig. S2). We found that the lung weight per body weight (%), concentrations of C3a and LDH concentration in the histone‐treated mice were significantly increased compared with those in the vehicle‐treated mice (Fig. 2A,C,D). In contrast, the expression of Crry/p65 was reduced in the histone‐treated mice, time‐dependently (Fig. S2). The association between Crry/p65 expression and lung oedema revealed a significant inverse correlation between the expression of Crry/p65 and lung weight per body weight (Fig. 2E; *R* = −0.708, *P* = 0.001). In addition, there was an inverse correlation between the expression of Crry/p65 and the concentration of C3a (Fig. 2F; *R* = −0.487, *P* = 0.040). The significant inverse correlation between the expression of Crry/p65 and LDH levels in plasma showed the association between Crry/p65 expression and cell damage (Fig. 2G; *R* = −0.596, *P* = 0.009). Furthermore, extracellular histones dose‐dependently decreased Crry/p65 expression and increased LDH levels (Fig. 3). Collectively, these results indicate that the expression of Crry/p65 is associated with the severity of pulmonary oedema via the production of C3a as an anaphylatoxin. This suggests that Crry/p65 and C3a were key molecules in histone‐induced ALI. To assess the potential utility of C3a as a therapeutic target for ALI, we administered a C3a receptor antagonist to ALI model mice and demonstrated that this pretreatment ameliorated lung haemorrhage and oedema (Fig. 4).

### EC damage induced by extracellular histones is associated with reduced expression of Crry/p65

**Fig. 5:**
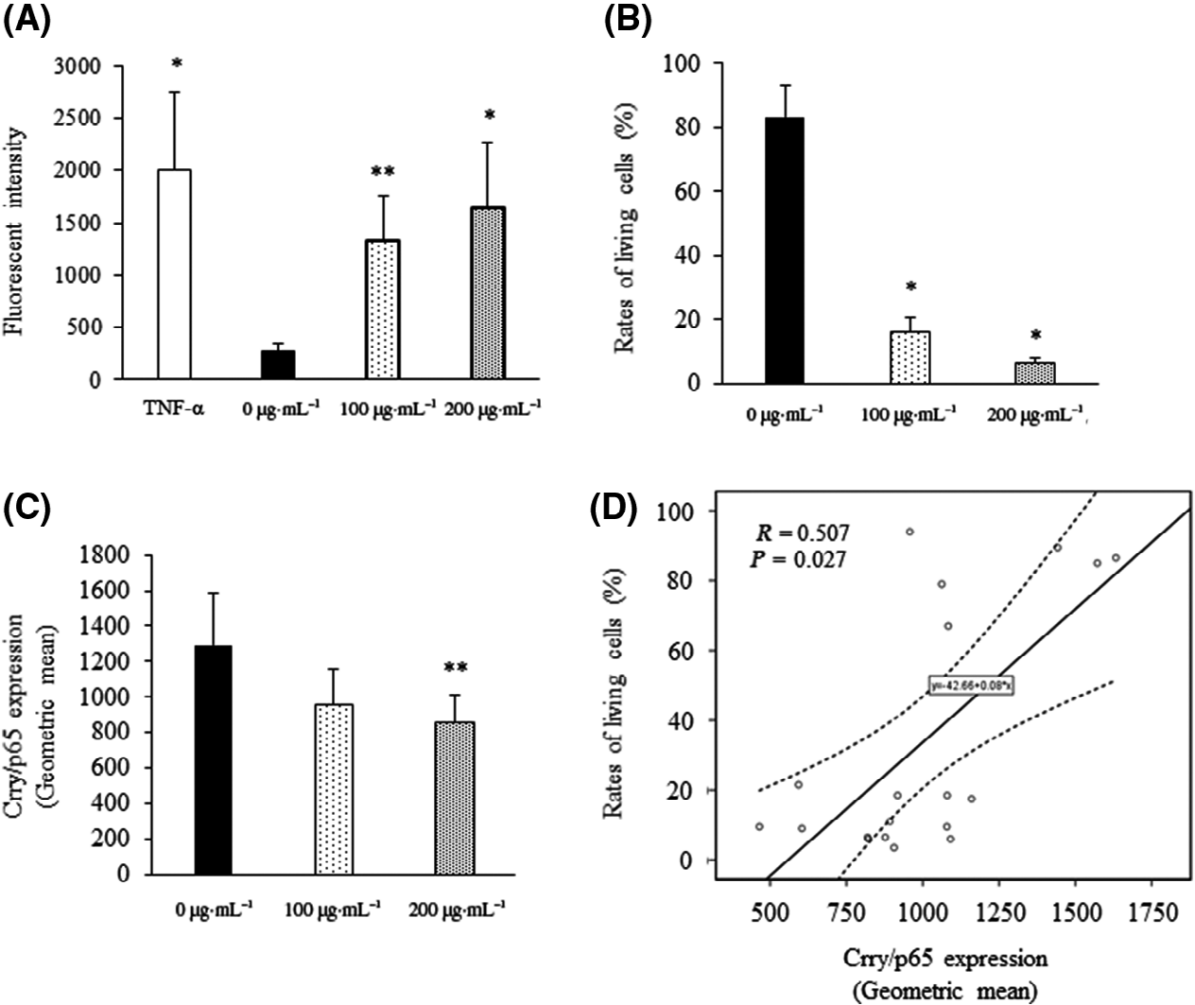
EC permeability and correlation between expression of Crry/p65 and proportion of living cells. Mouse vascular ECs were treated with calf thymus histones (0–200 µg·mL^−1^) for 30 min or mouse TNF‐α (positive control) for 19 h (*n* = 6 per group). Permeability of ECs was evaluated by fluorescent intensity shown in panel A. Rate of living cells and Crry/p65 expression are shown in panels B and C, respectively. Panel D shows the correlation between the rates of living cells and Crry/p65 expression. Values are shown as experimental means ± SD. **P < 0.05, *P < 0.01 vs. 0 µg·mL^−1^ (Tukey's test).

In the present study, we investigated whether endothelial damage was linked to a reduction in the expression of Crry/p65 by measuring the percentage of living cells and expression of Crry/p65 in response to exposure to extracellular histones. We observed dose‐dependent decreases in both the proportion of living cells and the expression of Crry/p65 (Fig. 5A,B). Moreover, we detected a significant correlation between the expression of Crry/p65 and the percentage of living cells (*R* = 0.507, *P* = 0.027). These results suggest that endothelial damage is associated with the impairment of Crry/p65.

### Extracellular histone exposure enhances C3 deposition

**Fig. 6:**
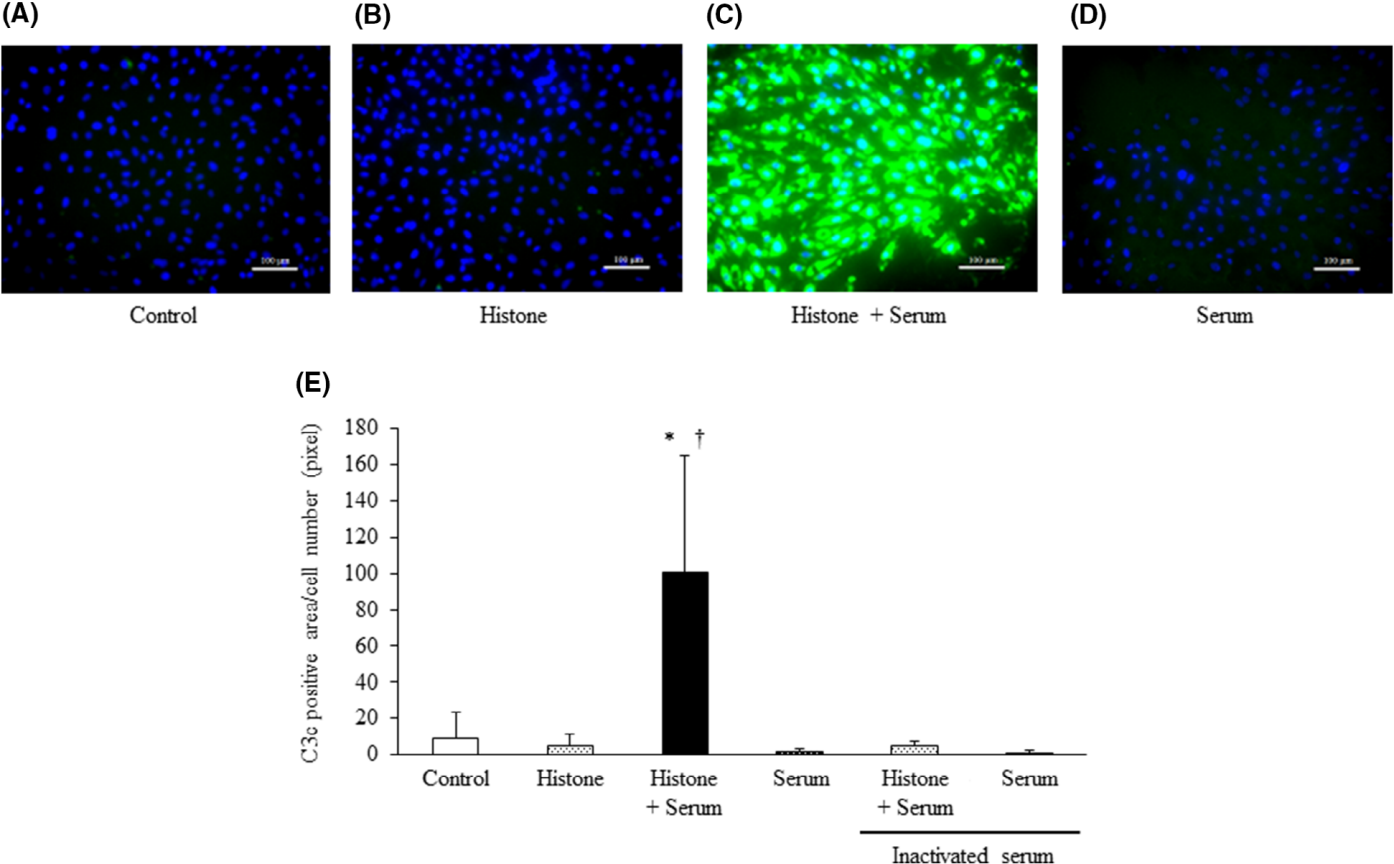
Treatment with extracellular histones promotes C3c deposition on the surface of vascular ECs. Representative images of C3c deposition are shown in panels A–D. Measurement of the C3c‐positive area and cell counts were performed in five randomly selected fields. Panel E shows the C3c‐positive area/cell number for each group (*n* = 6 per group). Values are shown as experimental means ± SD. *P < 0.01 vs. control, ^†^ *P* < 0.01 vs. serum (Tukey's test).

We next investigated whether a reduction in the expression of Crry/p65 was related to functional disorders in C regulation. We found that C3c deposition was significantly increased in histone‐treated RCB1994 cells incubated in medium containing normal serum (Fig. 6C,E). This increase was not detected when heat‐inactivated serum was used, thereby indicating that a functional disorder of Crry/p65 was induced by exposure to extracellular histones.

## Discussion

Crry/p65 is a protein expressed on the surface of vascular ECs that plays a role in protecting host cells from abnormal C activity in blood vessels. The surface of these cells contains glycoprotein molecules bearing sugar chains that function in the control of coagulation. Therefore, vascular EC damage induced by extracellular histones might trigger abnormal C activity and disrupt coagulation, which can result in hypoxic respiratory failure and the development of multiple organ failure [30, 31, 32, 33, 34, 35, 36]. We have previously reported that chondroitin sulfate prevented coagulation disorder induced by extracellular histones [27]. Currently, however, the mechanisms underlying the association between endothelial damage and C activity remain unclear. To elucidate these mechanisms in ALI, we investigated the role of Crry/p65 in a corresponding animal model.

In the present study, the expression of Crry/p65 in mouse lung vascular ECs was reduced in response to extracellular histone exposure, and this decrease correlated with an increase in lung weight. We also observed that the serum concentration of C3a was increased in ALI model mice and that the concentration of C3a inversely correlated with the expression of Crry/p65 in lung vascular ECs. These results indicate that extracellular histones induce a decrease in Crry/p65 expression, which in turn leads to an increase in C3a production that could promote lung oedema.

Extracellular histones inhibit C activation by interaction with C4 [37]; therefore, histones might not promote the production of C3a directly. As C3a is produced by cleavage of C3 resulting from C activity, extracellular histones may promote the production of C3a through the impairment of Crry/p65. Our result suggests that histone directly promotes vascular hyperpermeability of ECs. In addition, C3a activates mast cells to release histamine, which promotes vascular hyperpermeability. Thus, we investigated whether a C3a receptor antagonist could ameliorate ALI and, indeed, found that this antagonist reduced the extent of pulmonary oedema and lung bleeding in our histone‐induced ALI animal model. These findings indicate that C3aR might represent a viable therapeutic target for ALI. Moreover, given that the C3a receptor antagonist does not directly inhibit the C pathway, the immunosuppressive effects of this treatment would be predicted to be less pronounced than those associated with other drugs. Hence, we propose that clinical applications of C3a receptor antagonists should be considered to evaluate the benefits of such drugs for patients with ALI.

Previous studies demonstrated that extracellular histones can damage ECs [8, 22, 23]. We also reported that the reduction in Crry/p65 was induced as a consequence of cell injury [24, 38]. We thus presumed that the decrease in Crry/p65 expression observed in the present study was induced by cell injury induced by extracellular histone. To verify this hypothesis, we measured cell viability and the expression of Crry/p65 after exposure to extracellular histones. We found that in response of ECs to incubation with histones, the resulting proportion of living cells correlated with the expression of Crry/p65, thereby providing support for our hypothesis.

In conclusion, the findings of the present study indicate that extracellular histones cause dysfunction of Crry/p65 and vascular EC damage. Furthermore, C3a could represent a viable therapeutic target for ALI.

## Conflict of interest

The authors declare no conflict of interest.

## Author contributions

FN and TM contributed to the study conception and design, performed the experiments, analysed and interpreted the data, and drafted the manuscript. MI, KT, NT and SM contributed to the study design and interpreted the data. MM contributed to the study conception and design, supervised the study execution and interpreted the data.

## Supporting information


**Fig. S1.** The macroscopic findings at 10–60 min after calf thymus histones administration. C57BL/6J mice received a single tail vein injection of calf thymus histones (45 μg·g^−1^ body weight) or saline. Lung samples were collected at 10 and 30 min after the injection of calf thymus histones.
**Fig. S2.** Expression of Crry on the ECs and lung tissue. Lung samples were collected from C57BL/6J mice (*n* = 6 per group). Blue, red, and green collars show nuclear, CD31, Crry, respectively. Microscopic findings are shown through haematoxylin and eosin stains. White and black bars show 100 μm. The expression of Crry/p65 of endothelial cells and CD31^+^Crry/p65^+^ area in the whole‐section were measured at 10, 30 min after the injection of calf thymus histones. Values are shown as experimental means ± SD. **P < 0.05 vs. vehicle, ^††^ *P* < 0.05 vs. 10 min histone (Tukey's test).

## Data Availability

The data that support the findings of this study are available from the corresponding author [tomohiro.mizuno@fujita-hu.ac.jp] upon reasonable request.
