# Supplementary material for: Expression of a Crry/p65 is reduced in acute lung injury induced by extracellular histones
Source: FEBS Open Bio. 2021 Nov 8;12(1):192–202. doi: 10.1002/2211-5463.13322 (PMC8727949; doi:10.1002/2211-5463.13322)
Supplement: Supplementary file 1 — Fig. S1. The macroscopic findings at 10–60 min after calf thymus histones administration. C57BL/6J mice received a single tail vein injection of calf thymus histones (45 μg·g−1 body weight) or saline. Lung samples were collected at 10 and 30 min after the injection of calf thymus histones. Fig. S2. Expression of Crry on the ECs and lung tissue. Lung samples were collected from C57BL/6J mice (n = 6 per group). Blue, red, and green collars show nuclear, CD31, Crry, respectively. Microscopic findings are shown through haematoxylin and eosin stains. White and black bars show 100 μm. The expression of Crry/p65 of endothelial cells and CD31+Crry/p65+ area in the whole‐section were measured at 10, 30 min after the injection of calf thymus histones. Values are shown as experimental means ± SD. **P < 0.05 vs. vehicle, †† P < 0.05 vs. 10 min histone (Tukey's test). [file FEB4-12-192-s001.docx]

**Supplementary Materials**


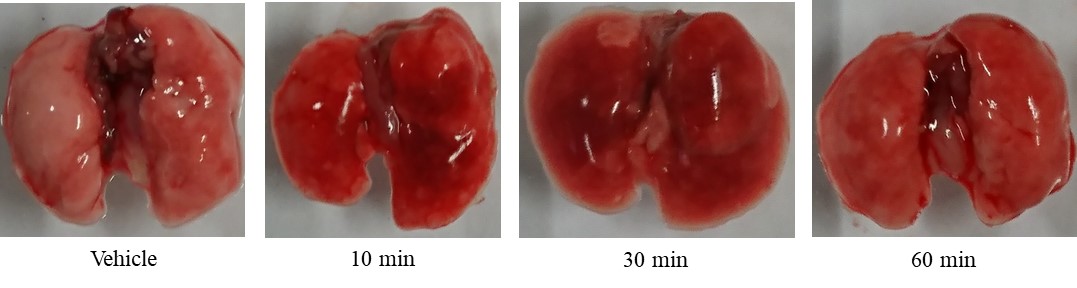


**Figure S1*.*** The macroscopic findings at 10 to 60 min after calf thymus histones administration.

C57BL/6J mice received a single tail vein injection of calf thymus histones (45 μg/g body weight) or saline. Lung samples were collected at 10 and 30 min after the injection of calf thymus histones.

**
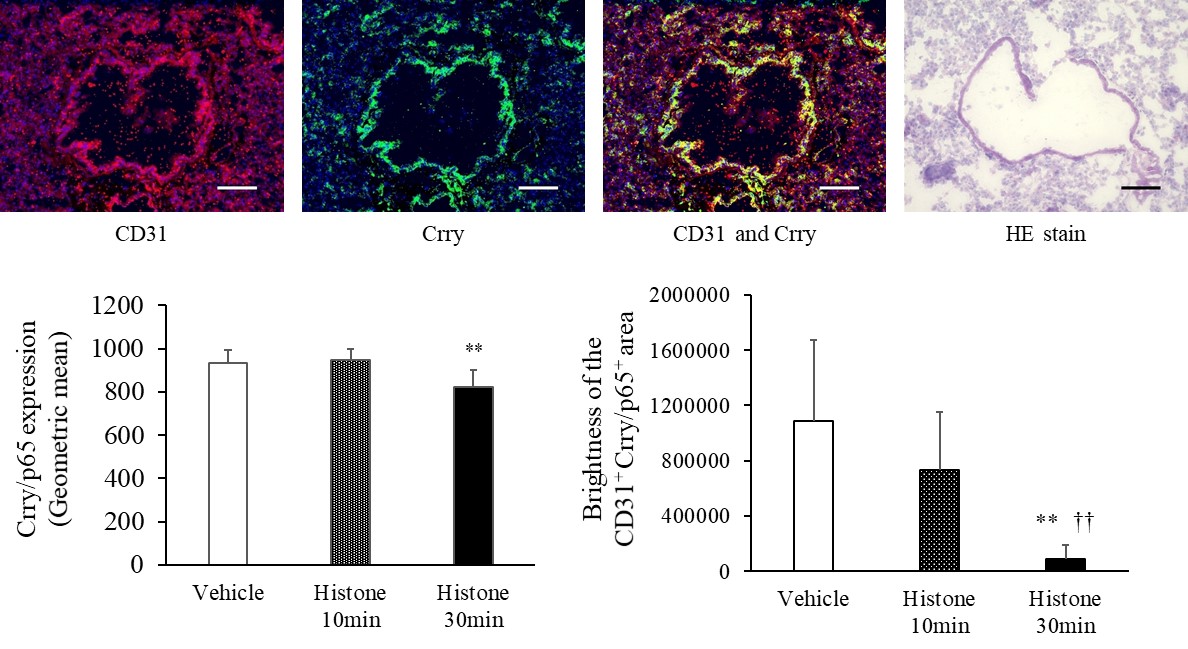
**

**Figure S2*.*** Expression of Crry on the endothelial cells and lung tissue.

Lung samples were collected from C57BL/6J mice (n = 6 per group). Blue, red, and green collars show nuclear, CD31, Crry, respectively. Microscopic findings are shown through haematoxylin and eosin stains. White and black bars show 100 μm. The expression of Crry/p65 of endothelial cells and CD31^+^Crry/p65^+^ area in the whole-section were measured at 10, 30 min after the injection of calf thymus histones. Values are shown as experimental means ± S.D. **p < 0.05 vs. vehicle, ^††^p < 0.05 vs. 10min histone (Tukey’s test).
